# Supplementary material for: Are carbon emissions trading and green financial instruments synergistic? -Comprehensive quantitative research based on content analysis
Source: PLoS One. 2024 Mar 7;19(3):e0298601. doi: 10.1371/journal.pone.0298601 (PMC10919601; doi:10.1371/journal.pone.0298601)
Supplement: S1 Data — (ZIP) [file pone.0298601.s001.zip › Data/地级市Z_F工作报告.zip!/╣≤╓▌/╫±╥σ╩╨/└┤╫╘╣1⁄2╓┌║┼í╛┬φ┐╦╩2╛▌═°í┐_2006.docx]

各位代表：

根据《中共遵义市委关于制定遵义市国民经济和社会发展第十一个五年规划的建议》，市人民政府制定了《遵义市国民经济和社会发展第十一个五年规划纲要（草案）》。现在，我代表市人民政府向大会作工作报告，请各位代表连同《纲要（草案）》一并审议，并请各位政协委员提出意见。

一、“十五”时期经济和社会发展回顾

“十五”时期，在省委、省政府及市委的正确领导下，在市人大及其常委会和市政协的监督支持下，我们高举邓小平理论和“三个代表”重要思想伟大旗帜，认真落实科学发展观，坚持以经济建设为中心，紧紧抓住国家实施西部大开发战略的历史机遇，强力推进工业化、城镇化和农业产业化进程，全面加强经济、政治、文化和社会建设，通过全市人民共同努力,“十五”计划顺利完成，西部大开发“五年打好基础”目标基本实现，经济社会发展取得重大成就。

——国民经济持续快速增长，综合经济实力跃上新的台阶。2005年，预计全市生产总值达404亿元，“十五”年均增长11.6%，比“九五”快1.5个百分点。人均生产总值达5437元，年均增长11.2%。农业结构调整和产业化经营取得新进展，粮食年产量基本稳定在300万吨左右，畜牧业、渔业产值占农业总产值的比重由2000年的31%提高到2005年的39%，“七大产业航母”已具雏形。工业化建设步伐加快，工业经济主导地位日益凸显。2005年，全市工业增加值预计达到131亿元，是“九五”期末的2倍，占生产总值的比重由“九五”期末的30.4%提高到34.1%；企业利润33.8亿元，是“九五”期末的3.2倍。第三产业开始提速，新型商贸形态不断涌现，旅游、房地产、通讯等新兴消费持续升温。经济结构调整取得重大进展，“十五”末，三次产业比重调整为25.1∶38.6∶36.3，实现了由“二一三”向“二三一”的重大转变。财税收入稳定增长，财政总收入由“九五”期末的24亿元增加到60.1亿元，年均增长20.2%；地方财政收入从11.6亿元增加到22.16亿元，年均增长13.8%；财政总收入占生产总值的比重由“九五”期末的10.3%提高到14.9%。

——基础设施建设力度加大，城镇化开创新的局面。“十五”期间，全社会固定资产投资完成678.5亿元，为“九五”期末的3.6倍，年均增长27.2%，有效拉动了经济增长。交通基础设施建设取得重大突破，“十五”期间，全市完成公路建设，提前实现了“县县通油路、90%的乡镇通油路、85%的行政村通公路”目标，公路通达深度和交通运输能力大幅度提高。以“西电东送”为重点的能源建设迈出重大步伐，初步形成“水火互济、大小并举”格局，装机容量达到319万千瓦，超过“十五”200万千瓦的预定目标。农业基础设施建设有所加强，农业灌溉、防灾抗灾、农村人畜饮水和城镇供水保障能力增强。生态建设成效显著，完成退耕还林357万亩，森林覆盖率达44%左右。“数字遵义”建设取得积极进展。城镇建设以前所未有的速度整体推进，产业、人口、生产要素加速向城镇集聚，城镇化率达31.9%，比“九五”期末提高了10个百分点。中心城区污水截流、饮用水源保护、小街小巷环境整治、10万亩风景林、植物园、红色旅游基础设施建设、旧城区改造、垃圾处理等工程顺利实施，城市功能日趋完善。城市管理力度加大，环境保护工作加强，人居环境不断改善，先后荣获“全国绿化模范城市”、“中国优秀旅游城市”、“国家园林城市”称号，创建湄潭县国家级生态示范区工作通过专家验收。以县城和中心集镇为重点的小城镇发展日新月异，新农村建设欣欣向荣，城乡面貌发生了巨大变化。

——科教兴遵和人才强市战略深入实施，各项社会事业取得新的进步。科技创新能力增强，科技成果转化加快，企业整体技术水平有新提高。基础教育进一步夯实，提前一年实现“两基”攻坚目标，“普九”人口覆盖率达100%；高中阶段毛入学率由“九五”期末的18%提高到28%，职业教育、成人教育、幼儿教育和特殊教育稳步推进；高等教育加快发展，遵义师范学院、遵义职业技术学院成功申办，合并组建遵义中西医药高等专科学校已经教育部专家组评审；素质教育全面推进，教育教学质量逐年提高；新建校舍面积175万平方米，教学条件明显改善。公共卫生服务体系框架基本形成，突发公共卫生事件应急处理能力有所增强，传染病发病率大幅度下降，成功防止“非典”疫情侵入。农村卫生“三项建设”和体制调整如期完成，城镇社区卫生服务快速发展，食品、药品安全工作明显加强。人口与计生工作继续保持全省领先位次，人口自增率由2000年的10.6‰下降到6.7‰，符合政策生育率从87.6%上升到94.94%。文化、广播电视基础设施建设进一步加强，广播、电视综合覆盖率分别达78.9%和87.5%。全民健身运动蓬勃开展，竞技体育水平不断提高，体育产业迈出新步伐。国防建设、人防战备和“双拥”工作不断加强，第五次荣获“全国双拥模范城”称号。民族宗教工作进一步加强，各民族团结和谐、共同发展。民政、外事、新闻、档案、妇女、儿童、老龄和残疾人等各项事业全面进步。

——城乡居民收入稳步增加，生活水平有新的提高。城镇居民人均可支配收入和农民人均纯收入分别达7982元和2319元，“十五”期间年均分别增长8.5%和5.6%，城乡居民储蓄存款余额年均增长8.5%。扶贫开发成效显著，五年脱贫24.55万人，贫困人口下降到12.6万人。五年新增城镇就业岗位15.7万个，城镇登记失业率控制在4.12%以内。社会保障体系进一步完善，“两个确保”全面落实，城镇职工养老、医疗和失业保险覆盖范围逐年扩大，工伤、生育保险相继启动；农村低保每人每月补差由13元提高到40元，农村计划生育家庭奖励扶助制度初步建立，新型农村合作医疗试点工作稳步推进。

——重大领域改革有所突破，开放型经济取得新的成效。国有经济结构战略性调整取得重大进展，市县属国有工业企业改革基本实现“两个100%”目标，国有资产监督管理体制进一步完善；所有制结构调整力度加大，非公有制经济成为国民经济新的增长点；农村税费制度改革成效显著，全面取消了农业税；行政审批制度、事业单位人事制度、社会保险制度、投资体制、财政体制、农村金融体制、社会事业、市政公用事业等各项改革有序推进。投资环境明显改善，开放水平进一步提高，“十五”期间招商引资到位资金166亿元。

——社会主义精神文明建设成绩喜人，依法治市取得新的进展。“四在农家”创建活动蓬勃开展，全市有10%以上的农户享受创建成果，“四民社区”创建工作有序推进，城乡精神文明建设水平整体提升。未成年人和大学生思想政治工作进一步加强，思想道德教育长效机制逐步建立。各级政府自觉接受人大法律监督、工作监督和政协民主监督，与各民主党派、工商联和群众团体的联系更加紧密；办理人大代表建议、批评、意见和政协委员提案及时率、满意率明显提高；市政府重大决策专家咨询、重大事项社会公示和听证等制度基本建立，政府决策民主化、科学化水平逐步提高；实施市政府新闻发布制度，群众的知情权、参与权和监督权得到有效保障；切实转变政府职能，改进工作作风，机关效能有所提高，依法行政的意识和能力明显增强；政府系统廉政建设不断加强，源头治理力度加大，政风明显好转；“平安遵义”创建工作深入推进，“四五”普法顺利完成，依法治市取得新成效；安全生产形势总体较好，全市保持安全稳定的社会环境。

“十五”时期是我市又一个发展比较快、效益比较好、群众得实惠最多的时期，为“十一五”加快发展，实现“新的飞跃”奠定了坚实基础，积累了基本经验：一是始终坚持以经济建设为中心，坚持发展第一要务，抢抓发展机遇，千方百计争上基础设施和优势产业重大项目，把扩大投资和促进消费作为驱动经济增长的关键，推动经济社会快速发展；二是始终坚定不移地实施“三化一强”战略，坚持以求真务实的作风，不断创新工作方式，完善配套政策措施，推动经济结构不断优化升级；三是始终坚持正确处理改革、发展和稳定的关系，着力构建社会和谐，营造稳定良好的经济社会发展环境；四是始终坚持统筹兼顾，致力于促进经济、政治、文化、社会全面协调和可持续发展；五是始终坚持以人为本，把实现好维护好发展好人民群众的根本利益作为各项工作的出发点和落脚点，认真研究解决人民群众生产生活中的突出问题，努力让人民群众分享改革发展的成果，提高人民生活水平。

各位代表，过去的五年，我们在克服困难和解决矛盾中前进，成绩来之不易，是全市人民和社会各界共同奋斗的结果。在此，我代表市人民政府向全市各族人民、知识分子、广大干部职工，向各民主党派、工商联、人民团体、无党派人士，向驻遵解放军指战员、民兵预备役人员、武警消防官兵、公安民警，向关心支持遵义经济社会发展的各界人士，表示衷心的感谢，并致以崇高的敬意！

在肯定成绩的同时，我们也要清醒地看到经济社会发展中还存在一些突出矛盾和问题。主要是：经济总量仍然偏小，人均水平较低；经济结构性矛盾突出，增长方式仍较粗放；投资力度仍需加大，基础设施瓶颈制约还未得到根本缓解；农民增收仍较缓慢，城乡居民收入差距拉大；社会保障体系不够完善，就业再就业压力较大；地方财政收支矛盾突出，政府偿债机制亟待健全等。这些问题，我们将在今后工作中努力加以解决。

二、“十一五”时期国民经济和社会发展的主要任务

“十一五”时期我市经济社会发展的指导思想：坚持以邓小平理论和“三个代表”重要思想为指导，坚持以科学发展观统领经济社会发展全局，坚持“立足科学发展，着力自主创新，完善体制机制，促进社会和谐”的方针，紧紧围绕最广大人民根本利益和全面推动经济、政治、文化和社会建设既快又好发展的根本要求，全面贯彻落实“六个必须”和“五个统筹”，着力创新发展模式、转变增长方式、调整经济结构、提高发展质量、推进“三化一强”，努力促进经济社会全面协调可持续发展和人的全面发展。

“十一五”时期我市经济社会发展的主要目标：生产总值年均增长12.5%以上，突破800亿元，人均生产总值达到1200美元以上；地方财政收入年均增长13%；全社会固定资产投资年均增长15%；社会消费品零售总额和外贸进出口总额年均分别增长16%和12%；到2010年三次产业结构调整为17∶43∶40；单位生产总值能源消耗比“十五”末降低20%左右；五年累计新增城镇就业15万人，城镇登记失业率控制在4.5%以内；人口自增率控制在 6.5‰以内；城镇居民人均可支配收入和农民人均纯收入年均分别增长8%和6%。为实现上述目标，今后五年要着力抓好以下工作：

（一）高度重视“三农”工作，努力建设社会主义新农村

继续把解决“三农”问题作为重中之重，坚持多予少取放活、工业反哺农业、城市支持农村的方针，统筹城乡发展，大力发展现代农业和非农产业，加快农村城镇化步伐，转移农村富余劳动力，农村总体实现小康，农业增加值年均增长5%以上。

——稳定提高农业综合生产能力。完成土地利用总体规划修编，加强基本农田保护；大力实施“三小”和骨干水利工程、烟（粮）水配套工程、沃土工程、坡改梯工程和土地治理工程，建设稳产高产基本农田，稳定提高农业生产能力；积极发展农机事业，提高农业装备水平。加强农业生态环境建设，巩固退耕还林成果，推进水土流失和石漠化综合治理，建立矿山地质环境恢复治理保证金制度，加强地质生态环境恢复治理工程建设；引导农民科学合理施用化肥、农药，切实防治耕地和水污染；加大农村沼气池建设力度，发展“四位一体”生态农业。

——扎实推进农业产业化经营。坚持以市场为导向，以农民为主体，运用市场机制，加大农业结构调整力度，稳定发展粮油，做大做强烟酒，加快发展畜、竹、药、椒、茶、蔬，着力打造“七大产业航母”，实施“六个一百”工程，走优质、高效、生态、安全、可持续的农业发展道路。围绕重点产业基地建设，培育壮大龙头企业，推动乡镇企业改造升级；加快发展农产品加工业，打造品牌，增强竞争力；发展农业经济合作组织，提高农民组织化程度；加强农业综合配套七大体系建设，逐步建立农产品市场准入和认证制度，积极推进农业标准化建设；实施农业科技增收工程，千方百计增加农民收入。

——努力建设社会主义新农村。按照“生产发展、生活宽裕、乡风文明、村容整洁、管理民主”的要求，以提高农业综合生产能力为重点，以农民增收为核心，有计划、有步骤、有重点地推进新农村建设。进一步丰富“四在农家”创建活动内涵，加大财政投入和社会帮扶力度，力争80%以上的农户享受创建成果。围绕解决“三个基本”问题，继续推进新阶段扶贫开发，加大集团帮扶、整村推进、易地扶贫和生态移民搬迁力度，集中扶持，重点帮助，抑制返贫，确保农村贫困人口稳定脱贫。全面完成农村危房改造，规划建设一批移民新村。加强农村交通、水利、供电、通讯、广播电视等基础设施建设，改善农村生产生活条件。加强乡村建设规划，积极整治镇村环境，提高农村文明程度。大力发展农村教育、卫生、文化等社会事业，促进农村经济社会协调发展。深入开展“三下乡”活动，充分发挥远程教育网络作用，丰富农民群众文化生活，全面提高农民思想道德、科学文化和健康素质，培养和造就新一代农民。

（二）加快新型工业化进程，强化工业经济的主导地位

坚持以信息化带动工业化，依托优势资源和骨干企业，以大项目和工业小区为载体，以技术进步为支撑，集中力量建成“六大基地”，培育“六个百亿”行业（企业）集团，推进“六大标志工程”建设，实现经济总量、结构和效益的新突破，工业增加值年均增长16%以上，占生产总值的比重达38%以上。

——加快发展优势产业。大力发展水火互济的能源工业，建成“西电东送”能源基地。建成构皮滩电站，力争开工建设习水二郎火电厂、桐梓火电厂和绥阳循环经济重化工基地自备电厂。抓好配套电煤基地建设，建成桐梓容光等7个百万吨级煤矿。到2010年，全市发电装机容量达1000万千瓦以上，煤炭年产量达到3000万吨，能源产业年销售收入达170亿元以上。支持遵义卷烟厂技改和茅台酒新增1万吨扩建，振兴地方白酒产业；培育壮大食用油、优质米、茶叶、辣椒、山野菜、肉类加工等龙头企业。到2010年，食品行业年销售收入达140亿元以上。加快遵义县80万吨/年氧化铝和务川氧化铝项目建设，实施遵义铝业股份公司新增30万吨电解铝等技改扩建项目，建设黔北循环经济型铝工业基地。到2010年，铝行业年销售收入达100亿元以上。加快遵义钛业、贵州钢绳、遵义双源化工、遵义瑞安水泥、南方镁业、众立化工等技改项目建设，建成国家级新材料产业化基地，到2010年，原材料行业年销售收入达100亿元以上。建成赤水20万吨竹浆纸一体化工程，加快建设绥阳循环经济型重化工、红花岗区深溪循环经济基地，抓好PVC等产品新技术的开发、引进，到2010年，化工行业年销售收入达150亿元以上。支持江南航天集团、长征电器集团、贵州海尔电器和天义电器有限公司加快结构调整和技术改造，到2010年，机电行业年销售收入达110亿元以上。加快发展以中药材深加工为主的地方特色药业，积极推进中药产业化，到2010年，制药工业总产值达20亿元以上。

——大力发展循环经济。坚持“减量化、再利用、资源化”的原则，科学规划，合理布局，逐步建立起以循环经济为主导的经济体系。以铝及铝加工、煤及煤化工为重点，推广热电、铝电、碱电等联产及燃气联合循环发电等资源综合利用技术；加快建设航天高科技、银河长征科技、遵义药业、海尔配套、湄潭绿色食品、赤水河谷名酒工业区，大力推进红花岗区坪桥新材料、桐梓煤化工、绥阳循环经济型重化工和遵义煤电铝一体化基地建设；积极开发和推广资源节约、替代和循环利用技术，大力发展资源消耗低、环境污染小、经济效益好的产业和企业，着力构建资源节约型和环境友好型社会。

——加速发展非公有制经济。认真贯彻“两个毫不动摇”的方针，努力消除影响非公有制经济发展的体制性障碍，放宽市场准入和投资领域，鼓励民间资金参与基础设施和公共事业建设。推动民营企业与同行业优势企业合作，促进各类所有制企业公平竞争，共同发展。改善金融服务，推进制度创新，促进非公有制经济向规模化、集约化转变。“十一五”期间，力争非公有制经济年均增长20%以上。

（三）培育壮大旅游产业，带动服务业加快发展

——做强做大旅游产业。抓住发展红色旅游机遇，完善旅游业发展规划，以“一点、一环、一线”为重点，把发展红色旅游同发展自然生态旅游、地域特色文化旅游、工业旅游、乡村旅游结合起来，把发展旅游产业同培育文化产业结合起来，实现旅游由观光型向避暑、休闲、渡假型转变。巩固“中国优秀旅游城市”创建成果，整合旅游资源，理顺管理体制，创新投资融资方式和市场运作机制，全力打造精品旅游景区和旅游线路。加大旅游合作和旅游宣传推介力度，打造川、黔、渝旅游“金三角”品牌。加强旅游基础设施和人才队伍建设，完善服务功能，提高服务水平和市场竞争力，努力把遵义建设成为红色旅游特色城市和生态旅游强市。到2010年，全市接待游客800万人次以上，实现旅游综合收入64亿元。

——加快发展现代服务业。积极发展生活消费型服务业，大力发展现代生产服务业；加快物流园区建设，培育大型流通企业和专业物流企业；改造提升传统服务业，规范发展现代服务业；抓好服务业重点项目建设，打造服务业知名品牌；完善消费政策，改善消费环境，促进消费增长。力争服务业年均增长15%，占生产总值的比重达40%以上。

（四）加速城镇化进程，促进区域协调发展

——大力推进城镇化。构筑以中心城区为核心、县城为支点、重点镇为结点、功能分区明晰、特色鲜明的城镇体系。到2010年，力争城镇化水平达40%以上。中心城区要继续按照“南北拓展、东西贯通、改造旧城、完善功能”的思路，开发建设忠庄、南白（龙坑）片区、董公寺和新蒲教育科技新区，构筑100万人口城市基础框架。抓好中心城区道路交通疏解工程建设，加大旧城改造和新区开发建设力度，完善城市功能分区，打造特色街区。县城要把旧城连片改造与拓展新区结合起来，推进“城中村”改造，适度拓展城区规模，形成一批10万人以上的中小城市，争取遵义县、桐梓、绥阳、湄潭撤县设市（区）。打造特色小城镇，重点扶持发展一批市场带动型、旅游带动型、工业带动型的中心集镇；按照建设中国酒文化名镇的思路，规划、建设、管理好茅台镇。加强城镇基础设施建设，不断完善城镇功能，改善人居环境。深化城镇建设投融资体制改革，建立多元化投融资体系。深化户籍、土地流转、就业、入学、社会保障制度改革，为城镇集聚人口创造条件。坚持建管并重，加大城市综合执法力度，加强城市管理，深化国家园林城市创建活动，力争建成国家卫生城市、全国文明城市和国家环保模范城市。

——促进区域协调发展。按照“紧贴黔中经济圈、融入长江经济带、参与泛珠经济区”的思路，加强对区域经济发展的导向和调控，统筹区域重大基础设施建设和生产力布局，推进资源整合和优势互补，着力建设中路出海大通道经济带、西部赤水河流域经济带、东部特色经济带。打破行政分割，健全区域经济合作开发的利益分配机制。继续支持经济强县强镇加快发展，形成一批经济总量上百亿强县和十亿强镇。进一步加大对贫困地区、民族地区经济社会发展的扶持力度，加快实施《务、正、道、凤片区开发规划》，促进区域经济协调发展。

（五）大力加强基础设施建设，为加快发展构筑支撑平台

——加强以公路为重点的综合交通运输体系建设。围绕构建“四纵三横三联二环”骨架公路网布局规划，突出抓好一批经济路、旅游路、资源路建设。建成扎佐至南白、白腊坎至茅台高速公路，力争建成杭瑞（遵义段）、遵义至福泉（遵义段）、仁怀至赤水、新蒲至新舟机场、遵义中心城区环线、仁怀至银水、赤水至泸州等高速和高等级公路，总里程达；建成绥阳至道真、湄潭至余庆、茅台至土城、凤冈经务川至道真、习水至桐梓、桐梓至容光等二级公路，总里程达；继续加强农村公路建设，建立健全农村公路养护管理长效机制，新建通村公路，沟通771个行政村。加快功能完善的汽车客、货运站（场）和物流中心建设。积极做好过境铁路项目的前期工作。 力争启用新舟机场、开工建设茅台机场。以乌江和赤水河航道整治为重点，加快港口、码头、农村渡口和构皮滩电站配套航运设施建设。

——加强水利基础设施建设。以解决工程性缺水为着力点，加快重点水利工程建设。完成遵义灌区一期等续建工程，力争建成湄凤余灌区二期、正安石峰水库灌区一期、道真大沙河水库、绥阳后水河灌区改造等一批重点水利工程。加大治理病险水库力度。积极推进蓄水节水工程建设，着力解决农村人畜饮水困难，全面启动农村饮水安全工程,新建“三小”工程40—60万个，人均有效灌溉面积达到0.5亩。继续加大农村小水电开发力度，大力实施县城供水管网改造、县城和乡镇防洪工程，深化水资源管理体制改革，提高水资源综合利用水平。

——加强信息化基础设施建设。加快建设 “数字遵义” ，大力推进信息技术在工业、农业和服务业等经济领域的广泛应用，积极发展以网络服务业和电子商务为重点的信息产业。加快电子政务和政务信息网络平台建设，提高政务服务水平，带动社会信息化。

（六）深入实施科教兴遵和人才强市战略，为加快发展提供智力支撑

——优先发展教育事业。深化教育改革，全面推进素质教育。按“双高”普九标准，巩固提高“两基”攻坚成果，“十一五”末初中阶段毛入学率达99%以上；着力推进高中阶段教育，高中阶段毛入学率达55%，力争中心城区、湄潭、余庆、仁怀、赤水、遵义县和其余县的县城基本普及高中阶段教育。以就业为导向，调整结构，整合资源，加快发展职业教育，把我市较大的人口压力转化为人力资源优势。提高遵义职业技术学院、航天职业技术学院办学水平，建设6所国家级重点职高和中专，完成三级师范向二级师范过渡。积极发展高等教育，支持遵义医学院加快发展，加强遵义师院、遵义电大的建设，组建遵义中西医药高等专科学校，筹建遵义大学。重视和支持民办教育，规范办学行为，促进各类教育协调发展。加强教师队伍建设，依法保障教育投入，继续改善办学条件，努力解决困难学生、进城务工人员子女入学问题，使所有孩子都能有书读、有学上。

——大力推进科技创新和应用。加大科学普及和科技推广力度，加强科技创新和应用能力建设，建立科技创新激励机制，强化企业技术创新的主体地位，加强高新技术产业化建设，推动军民两用技术产业成长。支持民营科技企业发展。加强遵义国家级新材料基地建设，实施重点科技项目，提升工业经济整体水平。围绕农业主导产业，创建农业科技示范园区。加强科技服务体系建设，整合科技资源，促进资本与技术融合。加强知识产权保护，鼓励企业实施专利、标准和名牌战略。加大科技投入，到2010年，全社会科技研发投入经费占生产总值的比例达1%以上。

——创新人才资源开发机制。认真组织实施人才“15851”工程，力争人才总量和质量有较大突破。深化干部人事制度改革，创新人才培养选拔任用机制，完善考核评价办法；全面贯彻《公务员法》，努力建设勤政廉政公务员队伍。创新专业技术人才和企业经营管理人才培养引进使用机制，着力培养学科带头人，重奖有突出贡献的专业技术人才和拔尖人才，加强职业经理人队伍建设，大力培养农村乡土实用人才。建立完善人才市场体系，搭建人才流动平台，实现人才结构和分布合理化。在全社会大力营造尊重个性、鼓励创新、信任理解的用人环境，民主活泼的学术氛围，和谐融洽的人际关系 ，使各类人才充满实现自身价值的快乐感、贡献社会的成就感和得到社会尊重的荣誉感。

（七）在更高层面推进新一轮改革开放，增强发展活力

——深化行政管理体制改革。推进政府机构改革，创新管理方式，精减机构，提高效能，降低行政成本。切实转变政府职能，在搞好经济调节的同时，强化市场监管、社会管理和公共服务职责。继续深化行政审批制度、行政执法体制等项改革，推进相对集中行政许可权试点工作。健全科学民主决策机制和行政监督机制，加快建立权责明确、行为规范、监督有效、保障有力的行政执法体系，全面推进依法行政。

——深化农村改革。稳定和完善以家庭承包经营为基础、统分结合的双层经营体制，推动土地使用权流转，推行适度规模经营，健全对被征地农民的合理补偿机制。完善农村税费改革各项配套措施，积极推进农村综合改革试点。健全村民自治制度，完善村务公开，整顿和规范村级财务管理，建立有效的农民负担监督管理制度，防止农民负担反弹。深化粮食流通体制改革，确保全市粮食供求平衡和粮价基本稳定。

——深化国有企业改革。加快国有企业股份制改造和公司制改革，完善法人治理结构，加快建立现代企业制度；继续做好国有企业政策性关闭破产、分离办社会职能和主辅分离工作；加快国有企业产权制度改革，完善国有资产监督管理体制，推进国有资产在流动中保值增值。

——深化财税金融体制改革。创新财源建设体制和机制，不断壮大财政实力；优化支出结构，确保重点支出；加快公共财政体系建设，努力促进县、区（市）间基本公共服务均等；继续推进部门预算、国库集中收付、政府采购、收支两条线管理和非税收入管理改革，加快收入分配制度改革，积极推进公务消费改革。理顺税收征管体制，加大税收征管力度；推行财政支出绩效评价和财政投资评审制度，提高财政资金使用效益；完善政府债务偿债机制，防范和化解财政风险。深化农村信用社改革，完善农村金融服务体系。大力支持金融监管，积极防范和化解金融风险。

——加快现代市场体系建设。积极营造公开、公平、公正的市场环境，推动各类要素市场建设，建立和完善统一开放、竞争有序的市场体系，充分发挥市场配置资源的基础性作用。建立健全资源开发价格形成机制，促进资源节约和环境保护。

——做大出口总量。加快转变贸易增长方式，优化对外贸易结构，积极扶持一批技术含量、产品质量和附加值高的产品出口；推动出口企业管理、质量、环境等国际标准认证，增强出口产品竞争力，力争进出口贸易总额年均增长12%。加强外贸队伍建设，组建遵义海关。

——着力改善投资软环境。加强“诚信遵义”建设，健全投诉处理机制，严格执行损害投资环境责任追究制度，改进工作作风，提高服务质量，努力营造规范透明的法制环境、廉洁高效的政务环境、公平诚信的市场环境、安全稳定的社会环境，把遵义建成各类投资者商务成本的盆地、政府服务的高地、投资收益的福地，成为有志者共谋大业、共创辉煌的理想热土。坚持内外资并举，促进内外资企业平等竞争，进一步扩大对内对外开放，力争全市招商引资到位资金年均增长15%。

（八）以人为本，建设和谐遵义

——建立完善加强社会管理的体制机制。按照“民主法治、公平正义、诚信友爱、充满活力、安定有序、人与自然和谐相处”的总体要求，加快构建党委领导、政府负责、社会协调、群众参与的社会管理格局，建设“和谐遵义”。

——千方百计扩大就业，完善社会保障体系。实施积极的就业政策，强化促进就业和再就业的措施，突出解决困难行业、困难地区、困难群体的再就业问题。加快劳动密集型企业发展，开发公益性、社会服务性岗位；认真落实小额担保贷款、减免税费等就业扶持政策，实施就业援助制度，完善就业服务体系；切实维护劳动者合法权益，建立和谐劳动关系；加强农村劳动力技能培训，扩大劳务输出。“十一五”期间，向非农产业和城镇转移农村富余劳动力50万。加快完善社会保障体系。巩固“两个确保”，健全养老、失业、医疗、工伤、生育等保险制度，逐步扩大社会保障覆盖面。到2010年，城镇职工基本养老保险、失业保险、基本医疗保险覆盖率分别达75%、60%和80%以上。继续做好城镇居民最低生活保障，认真解决进城务工人员社会保障问题。建立健全农村特困群众、失地农民最低生活保障制度，完善农村社会保障体系和城乡一体化社会救助体系，加快经济适用房和廉租房建设，实现“居者有其屋”。

——控制人口数量，提高人口素质。建立和完善“依法管理、村（居）民自治、优质服务、政策推动、综合治理”的人口和计划生育新机制，进一步稳定低生育水平。全面开展计生优质服务，推进优质服务先进县创建活动；大力实施出生缺陷干预工程，综合治理人口性别比偏高问题，加强对流动人口的计划生育管理和服务。建立健全计划生育利益导向机制和社会保障体系。加快卫生事业发展，加强以乡镇卫生院为重点的农村卫生基础设施建设；加强城乡公共卫生体系建设，基本建立起适应经济社会发展和群众健康需求的突发公共卫生事件应急处理体系、疾病预防控制体系和卫生执法监督体系；加强妇幼保健工作，健全市级妇幼保健机构；全面推行新型农村合作医疗制度，大力发展和规范城镇社区卫生服务网络，着力解决群众看病难、看病贵问题。加大城乡体育基础设施建设力度，加速体育社会化、产业化进程。

——加强环境保护，促进人与自然和谐。严格执行环境影响评价制度，推行投资项目环保一票否决制，坚决防止先污染、后治理，严格控制发生新的污染和生态破坏。提高环境监管能力，加大环保执法力度，强化重点流域、区域和行业污染防治，确保污染物排放达标。加大工业污染治理力度，改善城乡生活能源结构，加强城乡饮用水源地保护，完善排污管网、垃圾集中处理等环保基础设施，建立健全污染事故应急预警机制。“十一五”期末，中心城区水、气、声环境功能区达到创建国家环保模范城市标准。各县城完成污水和生活垃圾处理设施建设，积极推进乡镇环保设施建设，实施小水电代燃料生态工程。加大林业生态和林业产业体系建设，继续实施好退耕还林和天然林保护工程，建成一批国家级生态示范区和赤水河流域300里竹廊。加强中心城区、县城和小城镇绿地生态系统建设，继续抓好公路、铁路、河流沿线绿化。加强自然保护区和森林公园建设。到2010年，全市森林覆盖率达48％以上。加大水土保持预防监督力度，严格控制人为水土流失，加强水资源规划和管理，强化矿产资源保护和开发，做好气候资源区划和利用，有效节约利用自然资源。加快建立生态效益补偿机制。

——加强安全体系建设，营造和谐稳定的社会环境。高度重视安全生产，强化安全生产责任制，继续以煤矿、交通、消防安全为重点，加大专项整治力度，消除安全隐患，坚决遏制重特大安全事故发生。实施“食品放心工程”，加强食品、药品安全整治，确保食品、药品安全。推进“平安遵义”建设，健全社会治安长效管理机制，加强公共场所、重点人群和重点区域的安全防范，提升打防控整体能力。扎实开展“五五”普法，进一步提高全民法律素质和全社会法治化水平。加强社会治安综合治理，依法严厉打击各种犯罪行为，努力维护社会稳定。建立社会利益协调机制，妥善处理各种社会利益关系，及时有效化解人民内部矛盾；继续做好清理拖欠工程款和农民工工资工作。认真落实信访责任制，坚持依法信访，维护信访秩序。加强防灾减灾能力建设和危机管理，完善应对突发事件、重大自然灾害的组织体制和应急机制。

（九）加强社会主义精神文明和民主政治建设

——加强社会主义精神文明建设。大力弘扬民族精神、时代精神和遵义会议精神，实施市民文明素质工程、文明城市创建工程和服务群众民心工程。加强社会主义思想道德建设，广泛开展文明社区、文明村镇、文明行业等各种形式的群众性精神文明创建活动，力争创建“全国文明城市”。积极推动学习型社会建设，构筑社会化、开放式、多层次的终身教育学习体系。培养哲学社会科学人才，加强重大理论问题的研究，繁荣哲学社会科学。以满足人民群众对文化产品的需求为目标，加强公共文化基础设施和体系建设，完善公共文化服务功能。加强对文物的保护和合理开发利用。加快公益性文化事业改革，推动经营性国有文化事业单位转企改制，初步形成以公有制为主体，多种所有制共同发展，充满生机活力的文化事业产业格局。新建遵义博物馆、四渡赤水战史陈列馆，筹建遵义群众文化活动中心、遵义出版社、遵义演出公司、遵义文物艺术品市场，力争建设红军长征影视文化基地。

——加强社会主义民主政治建设。各级政府要自觉接受人大及其常委会的法律监督、工作监督和政协、各民主党派的民主监督，认真及时办理人大代表建议、批评、意见和政协委员提案。健全行政决策社会公示、社会听证、专家决策咨询和新闻发布等制度，提高决策的科学化、民主化水平，保障人民群众的知情权、参与权和监督权。坚持和完善民族区域自治制度，促进各民族共同进步。发展基层民主，深入推进村务、政务、厂务公开，完善政务服务中心运行机制。支持深化司法体制改革，促进司法公正，完善法律援助制度。严格控制各种检查、评比、达标活动，切实降低行政成本，提高行政效能。强化行政执法监督，规范政府行政行为，全面推进依法行政。按照建立健全教育、制度、监督并重的惩治和预防腐败体系的要求，加强行政监察和审计监督，加大从源头预防和惩治腐败力度，加强政风、行风建设，着力解决人民群众最关心、最直接、最现实的利益问题，取信于民。坚持国防建设与经济建设协调发展，加强人民防空、交通战备、国防后备力量建设和国防教育工作，增强全民国防意识。

三、认真做好“十一五”开局工作

2006年是“十一五”规划的开局之年，开好头、起好步至关重要。2006年经济社会发展的主要预期目标是：地区生产总值增长12 .6%；全社会固定资产投资增长20%；社会消费品零售总额增长18%；财政总收入和地方财政收入分别增长17%和13%；城镇居民可支配收入增长8%，农民人均纯收入增长7%；城镇登记失业率和人口自增率分别控制在4.6%和6.7‰以内。

围绕上述目标，2006年着重抓好八项工作：一是扎实推进社会主义新农村建设。粮食产量完成320万吨，烟叶收购174万担，大力推进农业结构调整和产业化经营，深入开展“四在农家”创建活动，促进农村三个文明协调发展。二是加快新型工业化进程。着力培育壮大优势支柱产业，加快发展循环经济，加强煤、电、油、运等生产要素的协调调度，确保规模工业增加值增长 18 %以上。三是加快城镇化进程。完成中心城区市政、生态建设投资10亿元，小城镇建设投资22亿元，城镇化率提高2个百分点。四是继续保持投资增长的强劲态势。完成固定资产投资240亿元；续建和开工建设公路，完成交通建设投资30亿元。五是着力扩大消费需求。启动“八个一”工程，培育壮大旅游业，带动第三产业加快发展。确保旅游综合收入达20亿元以上，第三产业增加值增长16%以上。六是在更高的层面推进新一轮改革开放。力争重点领域的改革有新突破，招商引资到位资金80亿元，非公有制经济增加值增长20%以上。七是加快构建“和谐遵义”。八是切实加强政府自身改革和建设。

认真办好十件实事。1、实施数字电视整体平移工程，建设广播电视“村村通”工程2060座；建设遵义博物馆和13个县、区（市）文化馆或图书馆；2、新增“四在农家”创建点800个以上，让14万农户、65万农民受益；3、新建小水窖10万口，改善14万农民生活用水，完成6万口生态沼气池建设；4、完成3个县计生指导站和30个乡镇计生服务站的新建改造，完成53个乡镇卫生院改扩建工程，在赤水市、仁怀市、遵义县、余庆县全面推行新型农村合作医疗制度，惠及200万农民；5、完成40所寄宿制学校建设，从2006年起，对农村义务教育阶段学生全部免收杂费，继续落实好对农村义务教育阶段贫困家庭学生提供免费课本和寄宿生活费补助政策；6、完成农村万户危房改造一期工程；7、全面完成中心城区小街小巷环境整治，建成10万平方米经济适用住房；8、完成5个县城和茅台城区供水管网改造；9、建设通乡油路、通村公路和57个乡镇客运站；10、新增城镇就业3.24万人，其中解决下岗失业人员再就业0.86万人。

2006年国民经济和社会发展任务十分繁重，对各级政府提出了新的更高的要求。一是要加强学习。认真贯彻落实十六届五中全会精神，以继续开展保持共产党员先进性教育活动为动力，加强理论武装和知识更新，创建学习型政府，提高驾驭社会主义市场经济和处理复杂事务的能力。二是要解放思想。坚持解放思想、实事求是、与时俱进的思想路线，大力弘扬遵义会议精神，进一步树立积极进取、开拓创新、艰苦奋斗和开明开放的意识，以新的机制、新的理念、新的办法，实现经济社会的超常规发展。三是要狠抓落实。按照“八个坚持、八个反对”的要求，进一步深入实际，转变作风，集中精力谋发展、抓大事、办实事、解难事。各级领导干部尤其是主要领导要身体力行，率先垂范，深入重点部位，抓住关键环节，突破薄弱环节，确保各项工作扎扎实实、卓有成效地推进。

各位代表，遵义正处在新的飞跃的前夕。我们面临新的形势，肩负新的使命。让我们紧密团结在以胡锦涛同志为总书记的党中央周围，高举邓小平理论和“三个代表”重要思想伟大旗帜，全面落实科学发展观，在中共遵义市委的领导下，以只争朝夕的精神，以敢为人先的勇气，以求真务实的作风，和衷共济，众志成城，为实现“十一五”规划目标和遵义经济社会发展的历史性跨越而努力奋斗！
